# Regioselective synthesis of 2-(bromomethyl)-5-aryl-thiophene derivatives *via* palladium (0) catalyzed suzuki cross-coupling reactions: as antithrombotic and haemolytically active molecules

**DOI:** 10.1186/s13065-014-0074-z

**Published:** 2014-12-17

**Authors:** Komal Rizwan, Muhammad Zubair, Nasir Rasool, Shaukat Ali, Ameer Fawad Zahoor, Usman Ali Rana, Salah Ud-Din Khan, Muhammad Shahid, Muhammad Zia-Ul-Haq, Hawa ZE Jaafar

**Affiliations:** Department of Chemistry, Government College University, Faisalabad, 38000 Pakistan; Sustainable Energy Technologies (SET) Center, College of Engineering, King Saud University, PO-Box − 800, Riyadh, 11421 Saudi Arabia; Department of Chemistry and Biochemistry, University of Agriculture, Faisalabad, 38040 Pakistan; The Patent Office, Karachi, Pakistan; Department of Crop Science, Faculty of Agriculture, 43400 UPM Serdang, Selangor Malaysia

**Keywords:** Thiophene, Palladium, Suzuki cross-coupling reactions, Heterocycles, Aryl boronic acid, Antithrombotic, Haemolytic, Cytotoxicity

## Abstract

**Background:**

It is seen that the regioselective functionalizations of halogenated heterocycles play an important role in the synthesis of several types of organic compounds. In this domain, the Suzuki-Miyaura reaction has emerged as a convenient way to build carbon-carbon bonds in synthesizing organic compounds. Some of the most important applications of these reactions can be seen in the synthesis of natural products, and in designing targeted pharmaceutical compounds. Herein, we present the regioselective synthesis of the novel series of 2-(bromomethyl)-5-aryl-thiophenes **3a-i,***via* Suzuki cross-coupling reactions of various aryl boronic acids with 2-bromo-5-(bromomethyl)thiophene **(2)**.

**Results:**

The synthesized compounds were screened for their haemolytic and antithrombolytic activities. The novel compounds **3f, 3i** showed highest 69.7, 33.6% haemolysis of blood cells, respectively. The antithrombolytic activity of the compounds was found to be within low to moderate against human blood clot. The compound **3i** showed potent clot lysis (31.5%).

**Conclusions:**

Considering these results, it is concluded that the synthesized compounds can be used as a promising source of therapeutic agents.

## Background

In present days, the synthesis of new and safe therapeutic agents is getting high importance in the field of medicinal science and pharmaceuticals. Most precisely, sulphur containing heterocycles are seen as the center of activity due to their widespread use in several important medicinal compounds. However, it is seen that the success of thiophene as an important moiety of medicinal agents led to the introduction of new therapeutic drugs. Substituted thiophene derivatives are well known for their chemotherapeutic applications. Many thiophene based heterocyclic compounds have shown versatile pharmacological activities such as antimicrobial [1,2], antiamoebic [3], antiparasitic, anticancer [4], antifolates, antipsychotic [5], diabetes mellitus [6], anticonvulsant [7], analgesic [8], antidepressant [9], antihistaminic, anticholinergic [10], antiallergic [11]. In addition, the cholesterol inhibition activity and as antagonist against many hormones releasing receptors has also been reported. In a yet different context, the thiophene based heterocyclic compounds has also been employed in formulizing computer printer’s ink and as a raw material for herbicides and pesticides [12]. Some of the recent studies showed that the thiophene containing compounds constitutes an important class of materials, which show intrinsic electronic properties such as luminescence, redox activity, non-linear optical chromism and electron transport [13-17]. The incidence of death due to thrombosis is higher in the world. Antithrombolytic activity of thiophene based compounds has been reported in literature [18,19].

In the synthesis of several types of organic compounds, the transition metal-catalyzed reactions are well known for the formation of new carbon–carbon (C–C) bonds. In this context, the Pd-catalyzed Suzuki–Miyaura coupling reaction [20] is one of the most efficient and unique method for the C–C bonds formation due to the requirement of mild reaction conditions, easily available environmentally safe organoboron compounds, high tolerance of functional groups and easy handling of the by-products [21-25].

The Suzuki–Miyaura cross-coupling reaction, which produces biaryls has proven to be the most important building blocks in organic synthesis owing to their industrial applications. We have previously reported the synthesis of arylthiophenes by regioselective Suzuki cross-coupling reactions and they were potentially studied as pharmaceutical agents [26,27].

There are few reports about the Suzuki Cross Coupling reaction of benzyl halides with different palladium catalysts under variable reaction conditions. Langle *et al*. [28] reported the Suzuki cross coupling reaction of unsymmetrical diarylmethanes, while Bandgar *et al*. [29] reported ligand free Suzuki cross coupling reactions of benzylic halides with aryl boronic acid. Molander and Elia [30] described the Suzuki-Miyaura cross-coupling Reactions of Benzyl halides with Potassium Aryltrifluoroborates. The cross-coupling of benzylic bromides with various aryl boronic acids have also been reported under microwave conditions [31].

For the first time, the present work focuses on the synthesis of various palladium (0) catalyzed Suzuki cross coupled derivatives of 2-bromo-5-(bromomethyl)thiophene, particularly with the aim to investigate their biological activities (Haemolytic and Antithrombolytic activities).

## Results and discussion

### Chemistry

We have investigated the Suzuki cross coupling reactions of 2-bromo-5-(bromomethyl)thiophene (**2**) with various aryl boronic acids under optimized conditions. To the best of our knowledge, no such work on the synthesis and biological activities of 2-(bromomethyl)-5-aryl-thiophenes (**3a–i)** has been reported to date.

**Scheme 1:**
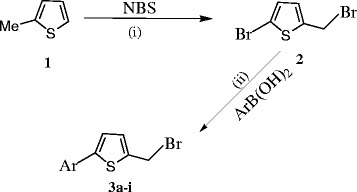
**Synthesis of intermediate compound 2-bromo-5-(bromomethyl)thiophene (2) and 2-(bromomethyl)-5-aryl-thiophenes 3a–i.** *Conditions: i,* **1**, (1 eq, 20.4 mmol), NBS (2.1 eq, 42.84 mmol), CCl_4_ (9–10 mL). *Procedure:* reflux **1** and NBS in CCl_4_ for 4–5 hours; *Condition ii*, **2** (1 eq, 0.976 mmol), Pd(PPh_3_)_4_ (2.5 mol%) aryl boronic acid (1.1 eq, 1.073 mmol), K_3_PO_4_ (2 eq, 1x.952 mmol), 1,4-dioxane/H_2_O (4:1) (Table 1), 12 h, 90°C.

As outlined in the reaction scheme (1), the first step in the synthesis of 2-(bromomethyl)-5-aryl-thiophenes (**3a–i)** is the preparation of intermediate compound 2-bromo-5-(bromomethyl)thiophene (**2**), which was obtained in 58% yield from the reaction between 2-methylthiophene (**1**) and N-bromosuccinamide in CCl_4_ [32] (Scheme 1).

In the next step, the Suzuki cross coupling reaction of appropriate aryl boronic acids with 2-bromo-5-(bromomethyl)thiophene (**2)** was carried out that eventually led to the corresponding 2-(bromomethyl)-5-aryl-thiophenes **(3a-i)** in moderate to excellent yields (25 − 76%) (Scheme 1, Table 1).

**Table 1:** **Synthesis of 2-(bromomethyl)-5-aryl-thiophenes (3a-i)**

**3**	**Aryl/ArylBoronic acids**	**Products**	**Solvent/H**_**2**_ **O (4:1)**	**Yield %**
a	3-Cl-4-F-C_6_H_3_		1,4-Dioxane	65
b	4-MeO-C_6_H_4_		1,4-Dioxane	76
c	4-Cl-C_6_H_4_		1,4-Dioxane	63
d	3,5-F_2_-C_6_H_3_		1,4-Dioxane	61
e	3-(MeCO)-C_6_H_4_		1,4-Dioxane	63
f	4-(MeS)-C_6_H_4_		1,4-Dioxane	56
g	4-I-C_6_H_4_		1,4-Dioxane	60
h	4-Me-C_6_H_4_		1,4-Dioxane	53
i	3,5-Me_2_C_6_H_3_		1,4-Dioxane	70

The Suzuki-Miyaura cross coupling reaction of benzyl halide with aryl boronic acid usually follow slow oxidative addition and facile reductive elimination as compared to aryl halide [33,34], therefore the reaction of aryl halide with aryl boronic acid is preferred over benzyl halide. The structures of these newly synthesized compounds were investigated from the data based on elemental analyses, Mass spectrometry, ^1^H-NMR and ^13^C-NMR spectra.

The results revealed that the compound 2-(bromomethyl)-5-(4 methoxyphenyl)thiophene **(3b)** was obtained in excellent yield (76%), which could be due to the solvent mixture (1,4-dioxane/water = 4:1), which has previously been reported to obtain high yields [35]. Another possible explanation is the high solubility of oxygen containing boronic acids in 1,4-dioxane, which led to the obtained high yield of compound **(3b)**. The coupling of intermediate compound (**2)** with 3-chloro-4-fluoro phenyl boronic acid also gave 2-(bromomethyl)-5-(3-chloro-4-fluorophenyl)thiophene **(3a)** in good yield. The products 2-(bromomethyl)-5-(4-chlorophenyl)thiophene **(3c)**, 2-(bromomethyl)-5-(3,5-difluorophenyl)thiophene **(3d)**, 1-(3-(5-(bromomethyl)thiophene-2-yl)phenyl)ethanone **(3e)**, 2-(bromomethyl)-5-(3,5-dimethylphenyl)thiophene **(3i)** were also obtained in relatively high yields ~ 63, 61, 63, 70% respectively. The obtained yield of products 2-(bromomethyl)-5-(4(methylthio)phenyl)thiophene **(3f)**, 2-(bromomethyl)-5-(4-iodophenyl)thiophene **(3g)**, 2-(bromomethyl)-5-*p*-tolylthiophene **(3h**) was fair as well (Table 1). In the cases, where low yield of products were obtained, the steric effects of substituents attached on aryl group of boronic acids and some practical problems associated with difficult chromatographic purification are suggested to be the possible issues [36]. Ortho-substituted aryl boronic acids have lack of reactivity and cannot couple in a good way due to steric factor. Hence, boronate anion of boronic acid are unable to attack easily on the substrate [36].

### Biology

#### Measurement of potential cytotoxicity by haemolytic activity

**Figure 1:**
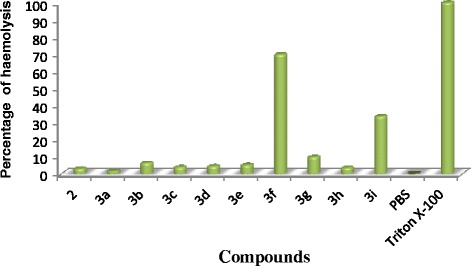
**Percentage of haemolysis of synthetic compounds 2, 3a-i.**

The cytotoxicity of the synthesized compounds *viz.***2 and 3a-i** was studied by examining the haemolytic activity against human red blood cells. The cytotoxicity of blood lymphocytes, thymocytes and spleen cells of various compounds are already known [37]. When compared with the positive control triton X-100 standard, the novel compounds **3f** and **3i** showed significantly high haemolytic activity ~ 69.7 and 33.6% lysis of blood cells respectively, which can be attributed to the presence of electron donating methyl groups. Molongi *et al*. [38] reported that the anticancer activity is often enhanced by the presence of electron releasing groups. In contrast, the compounds *viz.***2, 3a, 3b, 3c, 3d, 3e, 3g** and **3h** exhibited haemolytic activity below 10% (Table 2, Figure 1). In view of the observed differences in the % lysis of RBC values, it is inferred that the electron withdrawing and electron donating nature of the substituent groups have an influence on the haemolytic activity of the compounds [39]. Moreover, the cytotoxicity of the compounds *viz.***3f** and **3i** can be optimized by making appropriate changes in the molecular structures for the purpose of their use as toxic compounds to control the uncontrolled proliferation of cells [38].

**Table 2:** **Cytotoxicity studies by Haemolytic activity of synthetic compounds 2 and 3a-i**

**Compounds**	**% of haemolysis**
**2**	3.06 ± 0.03
**3a**	1.63 ± 0.02
**3b**	6.31 ± 0.07
**3c**	3.88 ± 0.04
**3d**	4.43 ± 0.05
**3e**	5.13 ± 0.05
**3f**	69.7 ± 1.23
**3 g**	9.87 ± 0.08
**3 h**	3.59 ± 0.03
**3i**	33.6 ± 0.87
**Phosphate-buffered saline (PBS)**	0.00 ± 0.00
**Triton X-100**	100 ± 0.58

The results are average ± S.D of triplicate experiments *p* < 0.05.

### Antithrombolytic activity

In the field of antithrombotic research, the compounds that exhibit anti-aggregatory activity have received a great deal of interest from research community. Several types of drugs such as heparin, ticlopidine, clopidogrel, plasminogen activator (t-PA), urokinase and streptokinase were explored as clot lysis agent but only few of them were found potent for clinical purposes [40-43].

**Figure 2:**
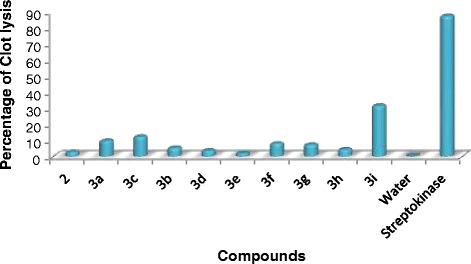
**Antithrombolytic activity of synthetic compounds 2, 3a-i.**

Because of high death rate due to cardio-vascular diseases, the clot lysis activity is very important characteristic of any drug [44,45]. The compounds under investigation exhibited low to moderate antithrombolytic activity against clot development in human blood. The compound **3i** showed potent clot lysis (~ 31.5%), whereas, the compounds **3e** and **2** showed comparatively low thrombolytic activity (Table 3, Figure 2). The values of % clot lysis for other compounds were found moderate. However, the results were significant *p* < 0.05, when compared with streptokinase taken as control. According to the best of our knowledge, no relative literature is available on this type of activity for such compound.

**Table 3:** **Percentage efficiency of Clot lysis of synthetic compounds 2 and 3a-i**

**Compounds**	**Clot lysis %**
**2**	2.73 ± 0.03
**3a**	9.76 ± 0.08
**3c**	12.3 ± 0.15
**3b**	5.29 ± 0.04
**3d**	3.71 ± 0.04
**3e**	1.96 ± 0.02
**3f**	8.05 ± 0.07
**3 g**	7.28 ± 0.06
**3 h**	4.37 ± 0.02
**3i**	31.5 ± 0.45
**Water**	0.43 ± 0.005
**Streptokinase**	87.2 ± 0.95

The results are average ± S.D of triplicate experiments *p* < 0.05.

## Experimental

### General

The melting points of compounds were determined using a Buchi melting point apparatus (B-540). High pure analytical grade reagents were used throughout all experiments and were purchased from Sigma Aldrich Chemical Co. (St. Louis, MO, USA) and Alfa Aesar Chemical Co. (St. Parkridge Ward Hill, MA, USA). ^1^H-NMR and ^13^C-NMR spectra were measured in CDCl_3_ and CD_3_OD (Bruker Aspect AM-300 instrument) at 300/75 MHz respectively. The chemical shift values (δ) were given in ppm and coupling constant was measured in Hertz (Hz)*.* EI-MS spectra were recorded on a JMS-HX-110 spectrometer with a data system. Elemental analysis was carried out by using CHNS/O analyzer (Perkin-Elmer 2400 series). For column chromatography, silica gel (70–230 mesh) and silica gel (230–400 mesh) were used. The reactions were monitored on TLC, using Merck silica gel 60 PF_254_ cards. The compounds were visualized by UV lamp (254–365 nm).

### General procedure for synthesis of 2-bromo-5-(bromomethyl)thiophene (2)

To carry out bromination of 2-methylthiophene, a weighed amount of starting material 2-methylthiophene (**1**, 1 eq, 20.4 mmol) suspended in 9–10 mL of dry carbon tetrachloride (CCl_4_) was made to react with N-bromosuccinimide (NBS) (2.1 eq, 42.84 mmol). This reaction mixture is then heated under reflux for four to five hours, followed by filtration and the removal of carbon tetrachloride under vacuum. Later on, the synthesized compound was purified by fractional distillation. Finally, the spectroscopic techniques were used to characterized the purified final product [32].

### General procedure for the preparation of 2-(bromomethyl)-5-aryl-thiophene (3a-i)

Pd(PPh_3_)_4_ (2.5 mol%) was added to 2-bromo-5-(bromomethyl)thiophene (**2**, 1 eq, 0.976 mmol) under nitrogen atmosphere and the resulting mixture was stirred for 30 min with the addition of 1,4-dioxane (2.5 mL). After 30 min the aryl boronic acid (1.1 eq, 1.073 mmol), K_3_PO_4_ (2 eq, 1.952 mmol) and water (0.625 mL) were added [27]. The whole mixture was stirred for 12 h at 90°C, and was later removed and cooled to room temperature. After cooling to ambient temperature, the mixture was diluted with ethyl acetate and the organic layer was separated, dried with magnesium sulfate and the solvent was removed under vacuum. The obtained crude residue was purified by column chromatography using ethyl-acetate and *n*-hexane in (1:1) ratio to get the desired products, which were further analyzed by using different spectroscopic techniques.

### Characterization data

*2-bromo-5-(bromomethyl)thiophene***(2)**. Brown liquid; ^1^H-NMR (300 MHz, CDCl_3_ + CD_3_OD): δ = 6.98 (d, *J* = 3.6 Hz, 1H), 6.83 (d, *J* = 3.6 Hz, 1H), 4.68 (s, 2H-CH_2_). ^13^C-NMR (75 MHz CDCl_3_ + CD_3_OD): δ = 25.4, 108.2 128.0, 128.8, 141.5; EI/MS m/z (%:) 255.0 [M^+**˙**^]; 257.0 [M^+^ (Br^79^, Br^81^) 100], 259.0 [M^+^ (Br^81^, Br^81^) 40], [M^+^-Br] = 177.0; [M^+^-CH_2_Br] = 163.0; [M^+^-2Br] = 125.8; [M^+^-Br and CH_2_Br fragment] = 84.0. Anal. Calcd. for C_5_H_4_Br_2_S: C, 23.46; H, 23.50. Found: C, 1.60; H, 1.60%.

*2-(bromomethyl)-5-(3-chloro-4-fluorophenyl)thiophene***(3a)**. Light yellow solid, Mp: 200-250°C; ^1^H-NMR (300 MHz, CDCl_3_ + CD_3_OD): δ = 7.54 (m, 1H-aryl), 7.52 (m, 1H-aryl), 7.36 (s, 1H-thiophene), 7.28 (m, 1H-aryl), 6.77 (s, 1H-thiophene), 4.07 (s, 2H-CH_2_). ^13^C-NMR (75 MHz CDCl_3_ + CD_3_OD): 27.4, 116.4, 121.2, 127.0, 127.6, 127.9, 129.0, 131.2, 137.0, 139.4, 158.0; EI/MS m/z (%): 304.8 [M^+**˙**^]; 306.8 [M^+^ (Br^81^,Cl^35^) 49]; 306.8 [M^+^ (Br^79^, Cl^37^) 17]; 308.8 [M^+^ (Br^81^, Cl^37^) 49]; [M^+^-F] = 287.2; [M^+^-Cl, F-fragments] = 253.1; [M^+^-CH_2_Br, thiophene, Cl- fragments] **=** 96.1. Anal. Calcd. For C_11_H_7_BrClFS: C, 43.23; H, 2.31. Found: C, 42.22; H, 2.40%.

*2-(bromomethyl)-5-(4-methoxyphenyl)thiophene***(3b)**. light yellow solid,Mp: 200-250°C; ^1^H-NMR (300 MHz, CDCl_3_ + CD_3_OD): δ = 7.12 (d, *J* = 8.4 Hz, 1H-thiophene), 6.84-6.81 (m, 4H-aryl), 6.52 (d, *J* = 3.6 Hz, 1H-thiophene), 3.98 (s, 2H-CH_2_), 2.03 (s, 3H-OMe). ^13^C-NMR (75 MHz CDCl_3_ + CD_3_OD): 20.4, 27.9, 125.4, 125.9, 127.7, 127.9, 129.0, 129.9, 130.0, 131.9, 135.0, 139.1; EI/MS m/z (%): 283.0 [M^+**˙**^]; 285.0 [M^+^ (Br^81^) 49]; [M^+^-CH_2_Br, thiophene] = 108.2; [M^+^-CH_2_Br, thiophene, OMe- fragments] = 78.0. Anal. Calcd. For C_12_H_11_BrOS: C, 50.90; H, 3.92. Found: C, 50.76; H, 3.80%.

*2-(bromomethyl)-5-(4-chlorophenyl)thiophene***(3c)**. Yellow liquid; ^1^H-NMR (300 MHz, CDCl_3_ + CD_3_OD): δ = 7.59 (d, *J* = 8.4 Hz, 2H-aryl), 7.40-7.37 (m, 2H-aryl, 1H-thiophene), 7.27 (s, 1H-thiophene), 4.70 (s, 2H-CH_2_). ^13^C-NMR (75 MHz CDCl_3_ + CD_3_OD): 27.6, 127.5 (2C), 128.5, 129.5 (2C), 128.4, 131.6, 134.1, 136.7, 139.1; EI/MS (m/z -ion mode): 286.9 [M^+**˙**^]; 288.9 [M^+^ (Br^81^,Cl^35^) 49]; 288.9 [M^+^ (Br^79^, Cl^37^)17]; 290.9 [M^+^ (Br^81^, Cl^37^) 49]; [M^+^-Br] = 223.0; [M^+^-CH_2_Br] = 194.2; [M^+^-CH_2_Br, Cl fragments] = 160.1, [M^+^-CH_2_Br, Cl, aryl fragments] = 83.2. Anal. Calcd. For C_11_H_8_BrClS: C, 45.94; H, 2.80. Found: C, 45.90; H, 2.70%.

*2-(bromomethyl)-5-(3,5-difluorophenyl)thiophene***(3d)**. Dark brown gummy matter; ^1^H-NMR (300 MHz, CDCl_3_ + CD_3_OD): δ = 7.42 (m, 1H-aryl), 7.22 (m, 1H-aryl), 7.12 (d, *J* = 2.4 Hz, 1H-thiophene) 6.55 (m, 1H-aryl), 6.51 (d, *J* = 3.6 Hz, 1H-thiophene), 4.51 (s, 2H-CH_2_). ^13^C-NMR (75 MHz CDCl_3_ + CD_3_OD): 27.5, 104.0, 111.1 (2C), 127.5 (2C), 136.1, 136.9, 140.0, 165.1 (2C). EI/MS m/z (%): 288.0 [M^+**˙**^]; 290.0 [M^+^ (Br^81^) 49]; [M-F] = 270.0; [M-2 F] = 254.2; [M-CH_2_Br] = 196.0; [M-CH_2_Br, thiophene fragments] = 113.1. Anal. Calcd. For C_11_H_7_BrF_2_S: C, 45.69; H, 2.44. Found: C, 45.75; H, 2.43%.

*1-(3-(5-(bromomethyl)thiophene-2-yl)phenyl)ethanone***(3e)**. Off white gummy matter; ^1^H-NMR (300 MHz, CDCl_3_ + CD_3_OD): δ = 7.87-7.85 (m, 2H-aryl), 7.50-7.41 (m, 2H-aryl), 6.91 (d, *J* = 3.6 Hz, 1H-thiophene), 6.66 (d, *J* = 3.6 Hz, 1H-thiophene), 4.83 (s, 2H-CH_2_), 2.57 (S, 3H-COMe). ^13^C-NMR (75 MHz CDCl_3_ + CD_3_OD): 26.5, 27.8, 126.0, 127.5 (2C), 128.5, 129.5, 130.5, 133.4, 136.0, 137.1, 139.0, 197.4. EI/MS m/z (%): 294.1 [M^+**˙**^]; 296.0 [M^+^ (Br^81^) 49]; [M^+^-Me] = 281.0 [M^+^-COMe] = 253.0, [M^+^-CH_2_Br] = 201.1, [M^+^-CH_2_Br, aryl, COMe] = 84.0. Anal. Calcd. For C_13_H_11_BrOS: C, 52.89; H, 3.76. Found: C, 52.80; H, 3.66%.

*2-(bromomethyl)-5-(4-(methylthio)phenyl)thiophene***(3f)**.off white solid,Mp: 110-150°C; ^1^H-NMR (300 MHz, CDCl_3_ + CD_3_OD): δ = 7.10 (m, 4H-aryl), 7.01 (d, *J* = 3.9, 1H-thiophene), 6.89 (d, *J* = 3.6, 1H-thiophene), 4.84 (s, 2H-CH_2_). ^13^C-NMR (75 MHz CDCl_3_ + CD_3_OD): 14.6, 27.8, 127.3 (2C), 127.5 (2C)127. 9 (2C), 130.0, 135.9, 139.4, 140.2. EI/MS m/z (%): 299.0 [M^+**˙**^]; 301.0 [M^+^ (Br^81^) 49]; [M^+^-Me] = 284.0; [M^+^-SMe] = 251.0; [M^+^-Br, Me fragments] = 219.0; [M^+^-CH_2_Br, thiophene fragments] = 109.1; [M^+^-CH_2_Br, thiophene, SMe fragments] = 77.5. Anal. Calcd. For C_12_H_11_BrS_2_: C, 48.16; H, 3.71. Found: C, 48.10; H, 3.77%.

*2-(bromomethyl)-5-(4-iodophenyl)thiophene***(3g)**. Brown solid, Mp: > 300°C; ^1^H-NMR (300 MHz, CDCl_3_ + CD_3_OD): δ = 7.76 (d, *J* = 2.1 Hz, 2H-Aryl), 7.58 (d, *J* = 1.8 Hz, 2H-aryl), 7.18 (d, *J* = 4.2 Hz, 1H-Thiophene), 6.82 (d, *J* = 3.3 Hz, 1H-Thiophene), 4.62 (s, 2H-CH_2_). ^13^C-NMR (75 MHz CDCl_3_ + CD_3_OD): 27.5, 94.6, 127.5 (2C), 129.0 (2C), 132.0, 135.7, 138.1 (2C), 140.0. EI/MS m/z (%): 378.1 [M^+**˙**^]; 380.0 [M^+^ (Br^81^) 49]; [M^+^-I] = 252.9; [M^+^-aryl, I] = 177.0; [M^+^-thiophene, aryl, I] = 95.8. Anal. Calcd. For C_11_H_8_BrIS: C, 34.85; H, 2.13. Found: C, 34.80; H, 2.11%.

*2-(bromomethyl)-5-p-tolylthiophene***(3h)**. Brown gummy matter;^1^H-NMR (300 MHz, CDCl_3_ + CD_3_OD): δ = 7.55-7.40 (m, 4H-aryl, 2H-thiophene), 4.68 (s, 2H), 2.35 (S, 3H-Me). ^13^C-NMR (75 MHz CDCl_3_ + CD_3_OD): 21.0, 28.1, 125.5 (2C), 127.5 (2C), 129.6 (2C), 130.5, 131.8, 136.5, 139.6. EI/MS m/z (%): 266.9 [M^+**˙**^]; 268.9 [M^+^ (Br^81^) 49]; [M^+^-CH_2_Br] = 175.0; [M^+^-CH_2_Br, Me fragments] = 161.0; [M^+^-CH_2_Br, thiophene fragments] = 91.0; [M^+^-CH_2_Br, aryl, Me fragments] = 83.4. Anal. Calcd. For C_12_H_11_BrS: C, 35.94; H, 4.15. Found: C, 35.84; H, 4.25%.

*2-(bromomethyl)-5-(3,5-dimethylphenyl)thiophene***(3i)**.Brown liquid; ^1^H-NMR (300 MHz, CDCl_3_ + CD_3_OD): δ = 7.24 (s, 1H-aryl), 7.14 (d, *J* = 9.9, 2H-aryl), 7.11 (d, *J* = 10.5 Hz, 1H-thiophene), 6.95 (s, 1H-thiophene), 4.97 (s, 2H-CH_2_); 2.35 (s, 6H-2Me). ^13^C-NMR (75 MHz CDCl_3_ + CD_3_OD): 21.8 (2C), 27.6, 127.4 (3C), 127.8, 130.8, 133.6, 135.9, 138.6 (2C), 139.8. EI/MS m/z (%): 280.2 [M^+**˙**^]; 282.2 [M^+^ (Br^81^) 49]; [M^+^-2Me] = 252.0; [M^+^-CH_2_Br] = 189.0; [M^+^-CH_2_Br, aryl, 2Me] = 83.2; [M^+^-CH_2_Br, Me]^ˉ^ = 174.0. Anal. Calcd. For C_13_H_13_BrS: C, 55.52; H, 4.66. Found: C, 55.58; H, 4.63%.

### Cytotoxicity studies by haemolytic assay

The cytotoxicity of synthesized compounds **2** and **3a-i** was determined by examining the haemolytic activity of human blood cells, following the previously reported method [46]. In a typical experiment, approximately 3 mL freshly obtained heparinized human blood was collected from the volunteers after consent and counseling. The blood samples were then centrifuged for 5 min at 1000 rpm, and the blood plasma was discarded and cells were washed three times with 5 mL chilled (4°C) sterile isotonic Phosphate-buffered saline (PBS) (pH 7.4). Erythrocytes were maintained 10^8^ cells per mL for each assay. Approximately, 100 μL of each synthesized compound was mixed with human blood cells (10^8^ cells/mL) separately. Later on, the Samples were incubated for 35 min at 37°C and agitated after 10 min. Soon after incubation, the samples were placed on ice for 5 min, followed by centrifuge for 5 min at 100 rpm. Supernatant 100 μL were taken from each tube and diluted 10 times with chilled (4°C) PBS. Triton X-100 (0.1% v/v) was taken as positive control and phosphate buffer saline (PBS) was taken as negative control and pass through the same process. The absorbance value was measured at 576 nm using μQuant (Bioteck, USA). Finally, the % RBCs lysis for each sample was calculated.

### Antithrombolytic activity

The blood samples were collected from volunteers after consent and counseling. Venous blood was pinched from healthy human volunteers without a history of anticoagulant treatment. The 100 μL of blood was transmitted to each of the previously weighed micro-centrifuge tubes to form clots. Then the solution of synthesized compounds **2** and **3a-i** (100 μL) having concentration of 1 mg/mL was added to the tubes, and incubated at 37°C for 45 minutes. Streptokinase was used as standard clot lysis agent and water as negative control for this assay. Clot lysis activity results were presented in percentage [47].

## Conclusions

In conclusion, novel series of 2-(bromomethyl)-5-aryl-thiophenes(**3a–i)** were synthesized, and the cytotoxicity of the newly synthesized compounds (**2, 3a-i)** against the human blood cells was investigated. Almost, all the tested compounds revealed some haemolytic activity in the safe range but in particular **3f and 3i** exhibited highest lysis of blood cells *viz.* 69.7 and 33.6 % respectively. The synthesized compounds exhibited low to moderate antithrombolytic activity against human blood clot. The compound **3i** was found more potent for clot lysis among all synthesized compounds. We anticipate that the continued investigation in this field will provide new insights and promote the progress towards the development of ideal thrombolytic therapy, characterized by maximized stable coronary arterial thrombolysis with minimal bleeding. The highly toxic compounds are deemed to be potential antitumor agents.
